# Sex-based differences in phagocyte metabolic profile in rats with monosodium glutamate-induced obesity

**DOI:** 10.1038/s41598-018-23664-0

**Published:** 2018-04-03

**Authors:** Mariia P. Rudyk, Valentyna V. Pozur, Daryna O. Voieikova, Yevheniia V. Hurmach, Nataliia M. Khranovska, Oksana V. Skachkova, Vitalina M. Svyatetska, Olexander G. Fedorchuk, Larysa M. Skivka, Tetiana V. Berehova, Liudmyla I. Ostapchenko

**Affiliations:** 1 Microbiology and Immunology Department, Educational and Scientific Centre “Institute of Biology and Medicine”, Taras Shevchenko National University of Kyiv, 2, Prospekt Hlushkov, Kyiv, 03022 Ukraine; 2 Department of Biochemistry, ESC “Institute of Biology and Medicine”, Taras Shevchenko National University of Kyiv, 2, Prospekt Hlushkov, Kyiv, 03022 Ukraine; 3 Research Laboratory of Experimental Oncology, National Cancer Institute, 33/43, Lomonosova str., Kyiv, 03022 Ukraine; 4 Research Laboratory of Microbiological and General Immunological Problems of Biotechnology, ESC “Institute of Biology and Medicine”, Taras Shevchenko National University of Kyiv, 2, Prospekt Hlushkov, Kyiv, 03022 Ukraine; 5 Department of Experimental Cancer Therapeutics, R. E. Kavetsky Institute of Experimental Pathology, Oncology and Radiobiology, National Academy of Sciences of Ukraine, Ukraine, 45, Vasylkivska Str., Kyiv, 22, 03022 Ukraine; 6 Research Laboratory of Pharmacology and Experimental Pathology, ESC “Institute of Biology and Medicine”, Taras Shevchenko National University of Kyiv, 2, Prospekt Hlushkov, Kyiv, 03022 Ukraine

## Abstract

The important component of obesity pathogenesis is inflammatory activation of innate immune cells within adipose tissue and in other body locations. Both the course of obesity and innate immune reactivity are characterized by sex-associated differences. The aim of the work was a comparative investigation of metabolic profiles of phagocytes from different locations in male and female rats with MSG-induced obesity. The administration of monosodium glutamate (MSG) caused obesity, with sex-associated differences, that was more severe in male rats. Obesity was associated with pro-inflammatory activation of CD14+ phagocytes from adipose tissue in female, but not in male rats, which was demonstrated by decreased phagocytosis activity along with increased ROS generation. Phagocytes from the peritoneal cavity and peripheral blood of obese female rats exhibited neutral metabolic profile, whereas those cells from obese male rats displayed a pro-inflammatory metabolic profile. Thus, the manifestation of obesity-induced inflammation was characterized by different patterns of metabolic profile of phagocytes in male and female rats. Identified immune cell characteristics expand our knowledge of obesity immunobiology and may help to develop more effective preventive and therapeutic interventions for obese patients of different sexes.

## Introduction

The worldwide prevalence of obesity and its metabolic complications have substantially increased in recent years^1,2^. The propensity towards development of obesity differs between the sexes, and this is, first of all, due to the effect of sex hormones on adipocyte metabolism^3,4^. In addition, sex-associated differences in cell types, other than adipocytes within adipose tissue, such as innate immune cells, also account for differences in obesity between males and females. Sex-based differences in immune responses are well documented. These differences are attributed to the immunomodulatory effects of sex hormones, as well as being related to the X chromosome gene contributions. The X chromosome encodes for a number of critical genes involved in the regulation of immunity, such as Toll-like receptors. Moreover, the X chromosome contains about 10% of all microRNAs in the genome, which regulate immune cell differentiation and functioning^5,6^. The sex differential expression of PRRs stipulates sex-specific activity of the innate immune cells following stimulation. Peritoneal phagocytes from female rodents produce higher levels of anti-inflammatory prostanoids, than do male-derived cells in response to microbial stimuli. Whereas, male phagocytes produce more pro-inflammatory cytokines and chemokines following PRR stimulation, than do female cells. The phagocytic activity of innate immune cells from many locations is higher in females than in males^7–9^. Sex hormones exert different immunomodulating effects. Natural level of testosterone shows a significant positive relationship with Th1 immune response, whereas natural level of estrogen – with Th2 immune cell metabolic shift^10,11^.

Adipose tissue and immune system are closely interrelated. Major alterations of immune responses expressed during obesity, have been represented as obesity-induced low-grade inflammation or «meta-inflammation»^12,13^. This disorder is associated with an increase in local adipose tissue of inflammatory cytokines and other proteins (TNFα, IL-1b, IL-6, IFNγ, MCP-1, iNOS) secretion, innate immune cell activation (adipose tissue infiltration by pro-inflammatory macrophages and neutrophils) as well as activation of pathogenic adaptive immune response^14,15^. The leading role in obesity-induced inflammation has been conferred on adipose tissue resident macrophages, as major inflammatory effector cells, whose number is increased in the fat, and producers of molecules that contributed to the inflammation. More recent studies have shown that adipose tissue resident macrophages express pro-inflammatory M1 (classically activated) phenotype during obesity, and they are closely related to the development of obesity-induced insulin resistance. In contrast, the fat from lean individuals contains mainly anti-inflammatory M2 (alternatively activated) macrophages, whose essential functions are debris phagocytosis and reparation, following the resolution of the inflammatory process^16–19^. An important role in obesity-induced inflammation is played by neutrophils as adipose tissue infiltrating cells and macrophage activators^20,21^. Although it is well established that obesity is associated with alterations of local (in adipose tissue) phagocyte metabolic profile, little is known about obesity-associated changes in phagocytes from other locations, which are involved in systemic meta-inflammation. Some authors have hypothesized that sex-associated differences in phagocyte metabolic polarization could play a role in the different disease incidence between males and females. Studies suggest that strategies to develop therapeutic interventions to treat obesity-associated diseases must take into account the differences in immune responses between males and females^22,23^.

Monosodium glutamate (MSG), which is known in the food industry as an umami taste substance, has been used for decades, not only in studies of diet-induced obesity, but also as a primary factor to induce obesity in animal models^24–27^. MSG administration in newborn animals causes injury to the ventromedial hypothalamic and arcuate nuclei. This leads to the development of obesity due to the lack of controlled balance between energy absorption and expenditure. Detailed mechanisms of this process have not been clearly understood^25^. A study on mice^27^ has shown sex- and strain-associated variations of metabolic and hormonal status during MSG-induced obesity.

In the present study, we performed a comparative investigation of metabolic profiles of phagocytes from different locations in male and female rats with MSG-induced obesity. We demonstrated that MSG-induced obesity development in rats is associated with the sex-dependent changes in the metabolic state, and the functional activity of monocytes and granulocytes located in adipose tissue, peritoneal cavity and peripheral circulation.

## Results

### White adipose tissue (WAT) measures in male and female rats with MSG-induced obesity

Neonatal treatment with MSG resulted in the onset of obesity in rats at the age of 4 months, and this was associated with hyperleptinemia and hyperinsulinemia, as described earlier^28^. The development of obesity was associated with slight diminution of animal body weight in male rats. Lee indices were also slightly increased only in male MSG-treated animals (Table 1). The administration of MSG during the neonatal period led to a significant gain of WAT depot weight in male and female rats (Table 1). Nevertheless, the total weight of WAT in obese male rats amounted to a quarter of animal body weight, whereas, in female rats, the relative weight of the total WAT was 10% of body weight. Sex-based differences in the distribution of WAT depots were also observed. The onset of obesity in female rats was characterized by a 2.5 fold increase of visceral fat pad weight, and the appearance of subcutaneous fat pads. No changes were registered in perigonadal WAT depots. In male rats injected with MSG, two-fold and 1.5 fold increase of visceral and perigonadal adipose tissue depots, correspondingly, was found, and the generation of large subcutaneous fat pads were registered (Table 1).

**Table 1:** Anthropometrical parameters and average white adipose tissue (WAT) weight in rats injected with monosodium glutamate (MSG).

Groups		Female rats (n = 8)		Male rats (n = 8)	
Parameters		Control animals	MSG-induced obesity	Control animals	MSG-induced obesity
**Weight, g**		212.4 ± 4.99	271.67 ± 3.36*	387.67 ± 6.25^##^	349.00 ± 24.28^##^
**Body length, cm**		18.71 ± 0.53	18.78 ± 0.51	24.75 ± 0.77^##^	20.75 ± 0.25*^##^
**Lee index**		0.317 ± 0.009	0.335 ± 0.006	0.295 ± 0.01	0.341 ± 0.006*
**Total WAT**	Absolute weight, g	7.8 ± 1.13	25.1 ± 6.7*	25.6 ± 2.06^##^	76.5 ± 4.07**^##^
	Relative weight, % body weight	3.1 ± 0.46	10 ± 2.3*	7.8 ± 0.19^##^	26.3 ± 1.4**^##^
**Subcutaneous WAT**	Absolute weight, g	Not detected	4.75 ± 1.35	Not detected	21.0 ± 4.46*^#^
	Relative weight, % body weight	Not detected	1.9 ± 0.5	Not detected	7.18 ± 1.4*^#^
**Visceral WAT** (including perigonadal adipose tissue)	Absolute weight, g	7.8 ± 1.13	20.35 ± 5.35*	25.6 ± 2.06^##^	55.5 ± 6.64**
	Relative weight, % body weight	3.1 ± 0.46	8.1 ± 1.9*	7.8 ± 0.19^##^	19.1 ± 2.3**
**Perigonadal adipose tissue**	Absolute weight, g	5.35 ± 1.85	5.15 ± 0.05	16.0 ± 1.4^##^	23.1 ± 2.58*
	Relative weight, % body weight	2.13 ± 0.77	2.07 ± 0.06	4.9 ± 0.27^##^	7.97 ± 0.91*^#^

Notes: Data are presented as mean ± SD. *P < 0.05 and **P < 0.001. χ² test was used for qualitative data. Comparisons between sexes (ANOVA) are shown as follows: ^#^p < 0.05, ^##^p < 0.01.

### Sex-dependent differences in the functional state and metabolic profile of adipose tissue infiltrating phagocytes

**Figure 1:**
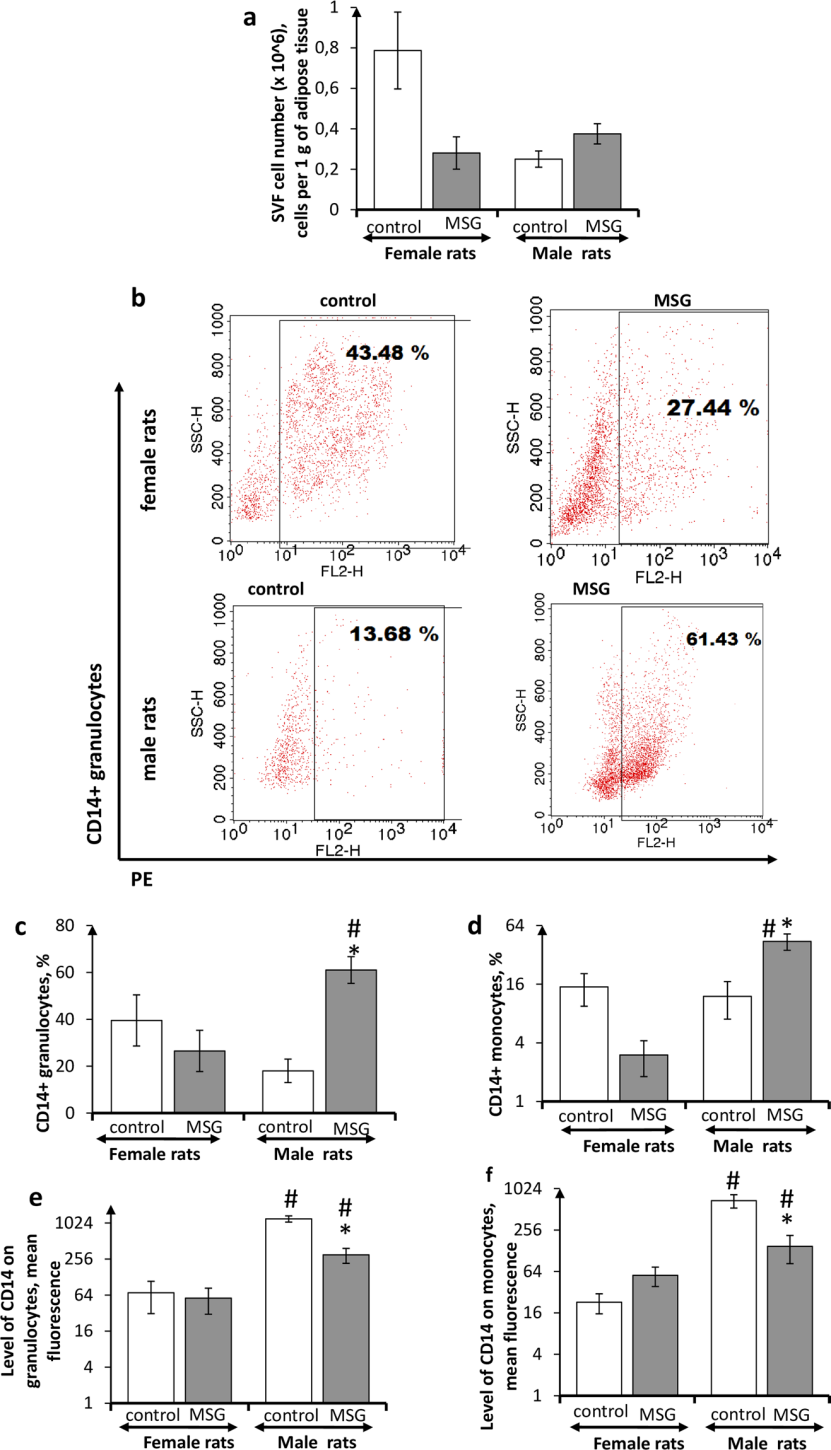
The number of CD14+ adipose tissue phagocytes in *female* and *male* rats with monosodium glutamate-induced obesity (MSG, n = 8). (**a**) SVF cell number; (**b**) flow cytometry representative plots for CD14+ granulocytes in female and male rats (PE staining), quantitations of CD14+ cells in stromal vascular fraction are shown; (**c**,**d**) number of CD14+ granulocytes and monocytes, respectively; (**e,f**) level of CD14 expression. Values in bar graphs are presented as mean ± SD. *P < 0.05 was considered significant, compared with the corresponding values of the control animal group. Comparisons between sexes (ANOVA) are shown as follows: ^#^p < 0.05. SVF stromal vascular fraction; PE Phycoerythrin.

Stromal vascular fraction (SVF) cells, in control animals, were characterized by the sex-dependent differences: the relative amount of SVF cells in visceral adipose tissue (including the perigonadal one) of female rats was 2.8 times higher than that in male rats (Fig. 1a). The development of MSG-induced obesity was associated with sex-dependent alteration in this index. A 2.8-fold decrease of SVF cell fraction was detected in obese female animals, whereas the number of SVF cells in obese male animals was 1.5 times higher than those in their non-obese counterparts.

Flow cytometry was used to differentiate CD14+ cells among the SVF cells isolated from adipose tissue and to determine metabolic profile of those CD14+ cells. Approximately an equal number of CD14+ phagocytes was revealed in SVF of control male and female animals (Fig. 1b–d). However, the level of expression of this marker on male rat SVF phagocytes was dozens of times higher than that of females (Fig. 1e,f). The development of MSG-induced obesity resulted in sex-dependent alterations in the frequency of CD14+ cells in SVF, and in the level of its expression. The number of CD14+ granulocytes and monocytes in obese female rats was lower than those in their non-obese counterparts, whereas both fractions of CD14+ phagocytes in obese male animals were more numerous than those in control male rats. The level of CD14 expression in SVF phagocytes from female obese rats did not differ significantly, as compared to the control group. In contrast, the expression of this phenotypic marker in male obese rats was 4 times lower than that in non-obese male animals.

**Figure 2:**
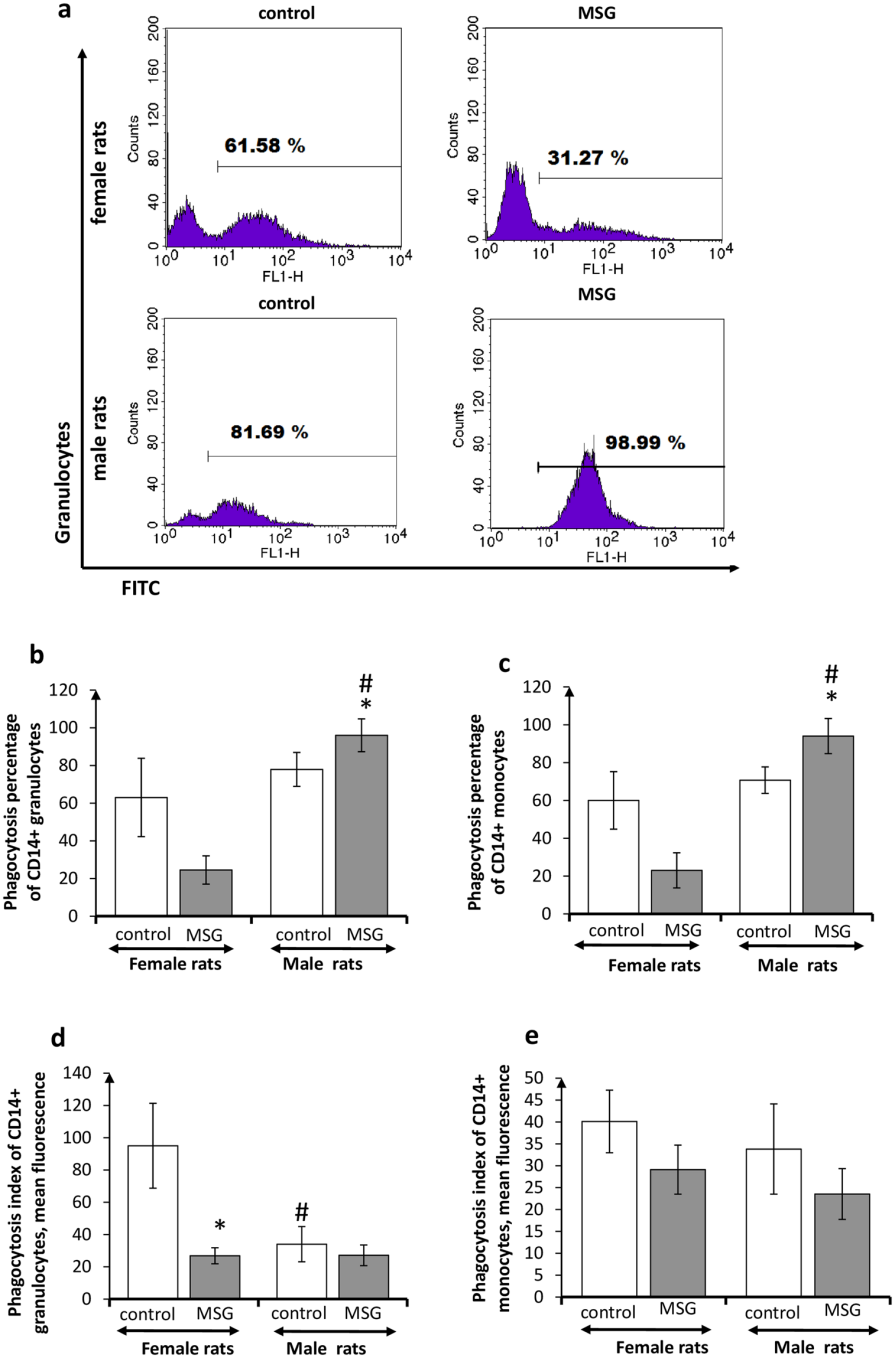
Phagocytosis activity of CD14+ adipose tissue phagocytes in *female* and *male* rats with monosodium glutamate-induced obesity (MSG, n = 8). (**a**) Flow cytometry representative histograms for CD14+ phagocytizing granulocytes in female and male rats (FITC staining), quantitations of phagocytizing CD14+ cells in stromal vascular fraction are shown; (**b,c**) phagocytosis percentage of CD14+ granulocytes and monocytes, respectively; (**d,e**) phagocytosis index of CD14+ granulocytes and monocytes, respectively. Values in bar graphs are presented as mean ± SD. *P < 0.05 was considered significant, compared with the corresponding values of the control animal group. Comparisons between sexes (ANOVA) are shown as follows: ^#^p < 0.05.

**Figure 3:**
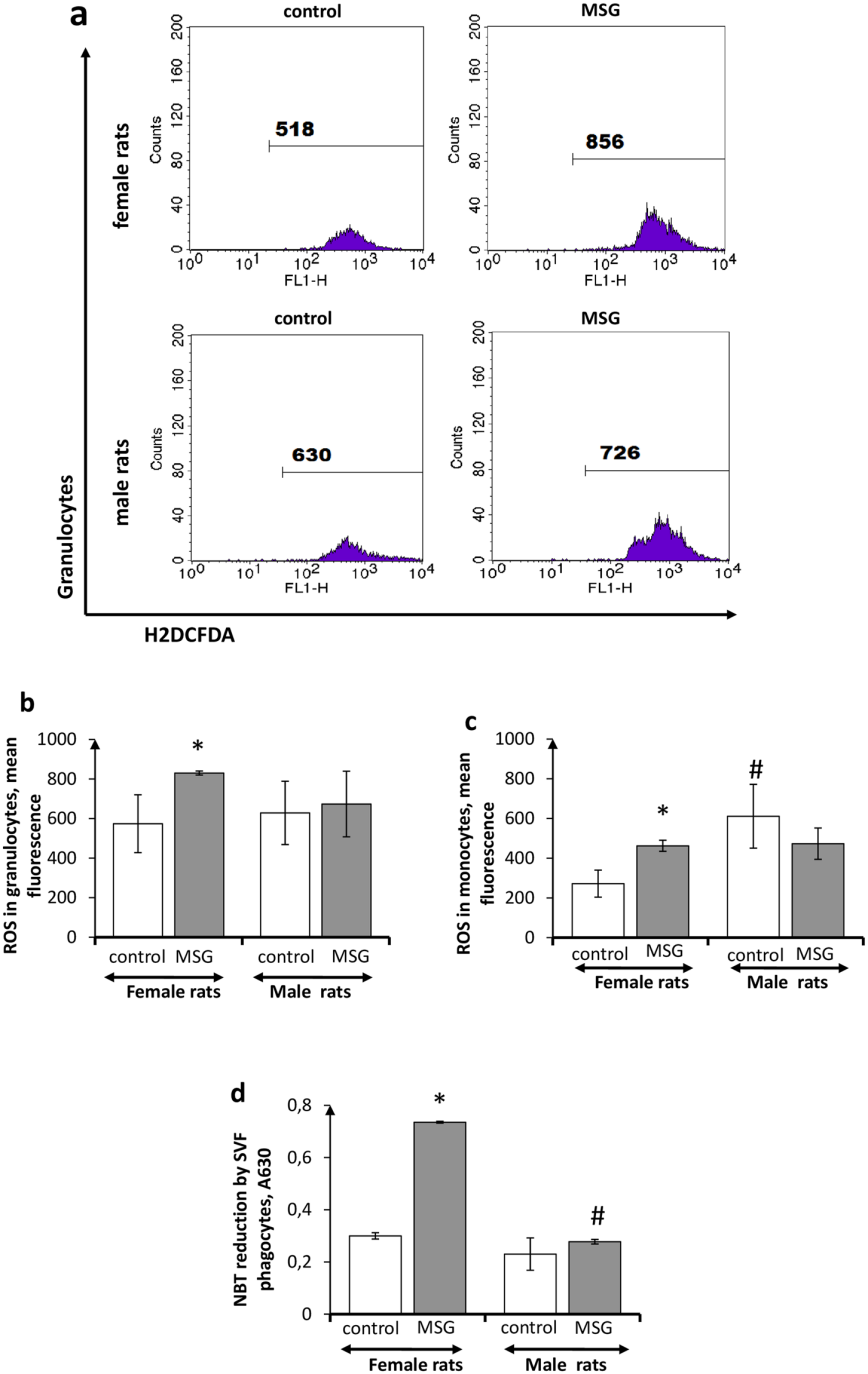
Oxidative metabolism of adipose tissue phagocytes in *female* and *male* rats with monosodium glutamate-induced obesity (MSG, n = 8). (**a**) Flow cytometry representative histograms for granulocytes in female and male rats (H2DCFDA staining), fluorescence intensity (ROS generation by cells in stromal vascular fraction) is shown; (**b,c**) intracellular ROS generation by granulocytes and monocytes, respectively; (**d**) extracellular ROS release (NBT-test) by stromal vascular fraction cells. Values in bar graphs are presented as mean ± SD. *P < 0.05 was considered significant compared with the corresponding values of the control animal group. Comparisons between sexes (ANOVA) are shown as follows: ^#^p < 0.05.

Phagocytosis parameters of SVF monocytes and granulocytes did not differ significantly in control male and female animals (Fig. 2). Radical differences in oxidative metabolism of SVF phagocytes were not observed either, especially in male rats (Fig. 3). Obesity, however, affected differently these indicators in females and males. The PhP and PhI of SVF phagocytes in females with obesity were slightly reduced, in comparison with non-obese females, whereas the oxidative metabolism of phagocytes from obese females was significantly enhanced, in comparison with that of the control rats (Figs 2 and 3). The quite different pattern of SVF phagocyte metabolism was observed in male rats with obesity. The SVF phagocyte PhPs in obese male animals were slightly raised, whereas PhIs and oxidative metabolism of SVF phagocytes did not differ significantly from those in control male rats.

### Sex-associated differences in peritoneal phagocyte metabolic profile in rats with MSG-induced obesity

**Figure 4:**
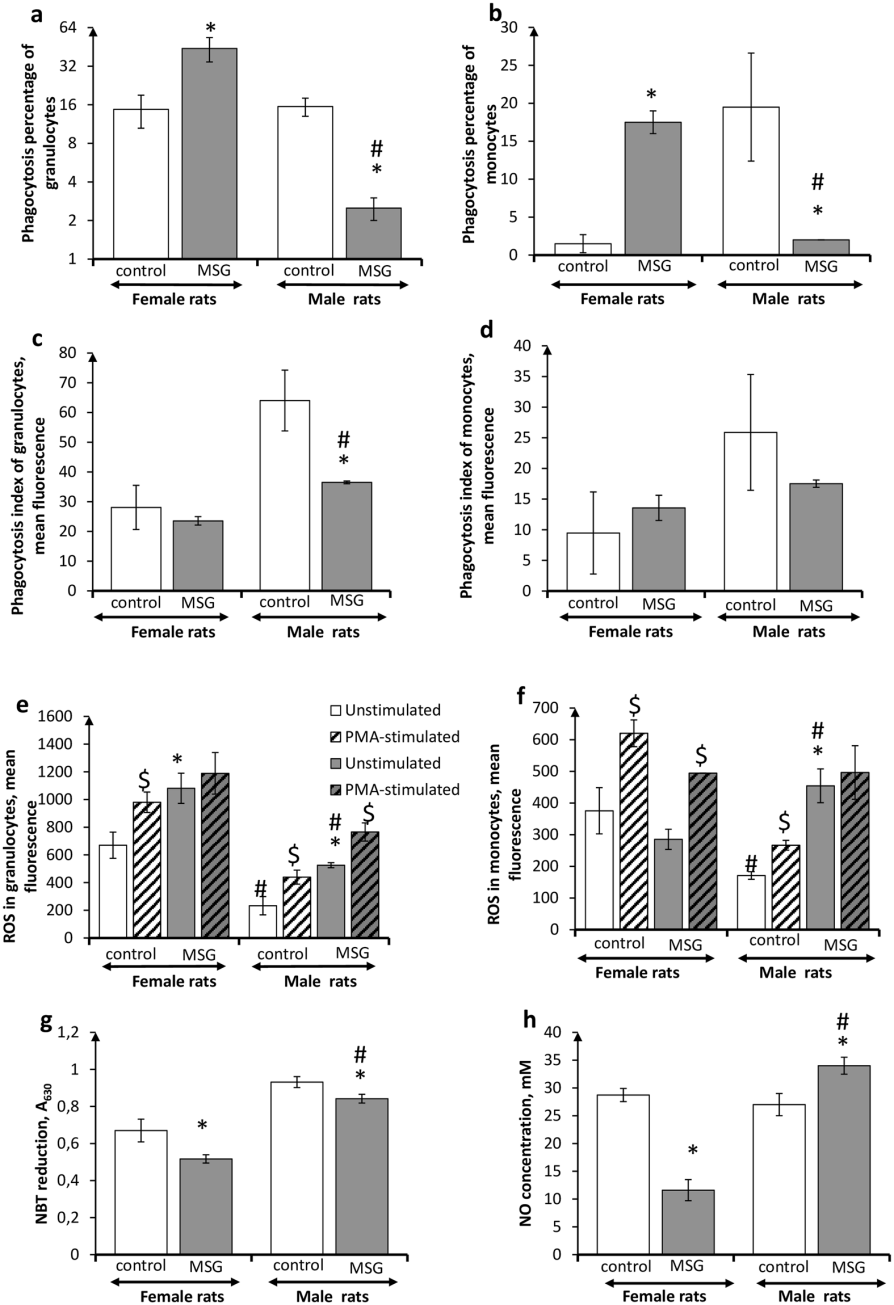
Functional activity of peritoneal phagocytes in *female* and *male* rats with monosodium glutamate-induced obesity (MSG, n = 8). (**a,b**) Phagocytosis percentage of granulocytes and monocytes, respectively; (**c,d**) phagocytosis index granulocytes and monocytes, respectively; (**e,f**) intracellular ROS generation by granulocytes and monocytes, respectively; (**g**) extracellular ROS release (NBT-test); (**h**) NO release (measured as nitrite level). Data are presented as mean ± SD. *P < 0.05 was considered significant compared to the corresponding values of the control animal group. ^$^P < 0.05 was considered significant, compared to the values of the corresponding unstimulated cells. Comparisons between sexes (ANOVA) are shown as follows: ^#^p < 0.05.

Sex-associated differences were observed in quantitative and functional characteristics of peritoneal cavity phagocytes in male and female control rats. The percentage of phagocytizing monocytes in peritoneal lavage from males was ten times higher than that in females (Fig. 4a,b). PhIs of peritoneal phagocytes from males were also significantly higher than those in females (Fig. 4c,d). Meanwhile, intracellular ROS-generation in peritoneal phagocytes was substantially lowered in male rats, as compared to females (Fig. 4e,f), although NBT reduction was moderately increased (Fig. 4g). Peritoneal phagocytes from control male and female animals positively responded to PMA *in vitro*. NO-generation had no sex differentiation (Fig. 4h).

MSG-induced obesity development led to sex-dependent changes in metabolic patterns of rat peritoneal phagocytes. PhPs of peritoneal phagocytes were significantly increased in obese females, but were profoundly decreased in obese males, as compared to their non-obese counterparts (Fig. 4a,b). PhI values in obese male and female animals were lower than those in control rats (Fig. 4c,d). Peritoneal granulocyte ROS generation was enhanced in obese male and female animals (Fig. 4e,f). However, female granulocytes lost the ability to respond positively to PMA *in vitro*, showing a maximal degree of activation, while male granulocytes did not. Treatment with PMA *in vitro* resulted in the stimulation of oxidative metabolism in granulocytes by 45%. Intracellular ROS generation of female peritoneal monocytes did not differ significantly from non-obese animals, whereas monocytes from obese male rats were characterized by the increased ROS generation, and the loss of a positive response to PMA *in vitro*, as compared to their non-obese counterparts. This indicated a maximal degree of activation of this function and an absence of oxidative metabolic reserve. In addition, peritoneal lavage phagocytes from female obese animals had lowered nitrite production, whereas phagocytes from obese male rats had slightly increased NO generation (Fig. 4h).

### Sex-associated features of circulating phagocyte metabolic profile in rats with MSG-induced obesity

**Figure 5:**
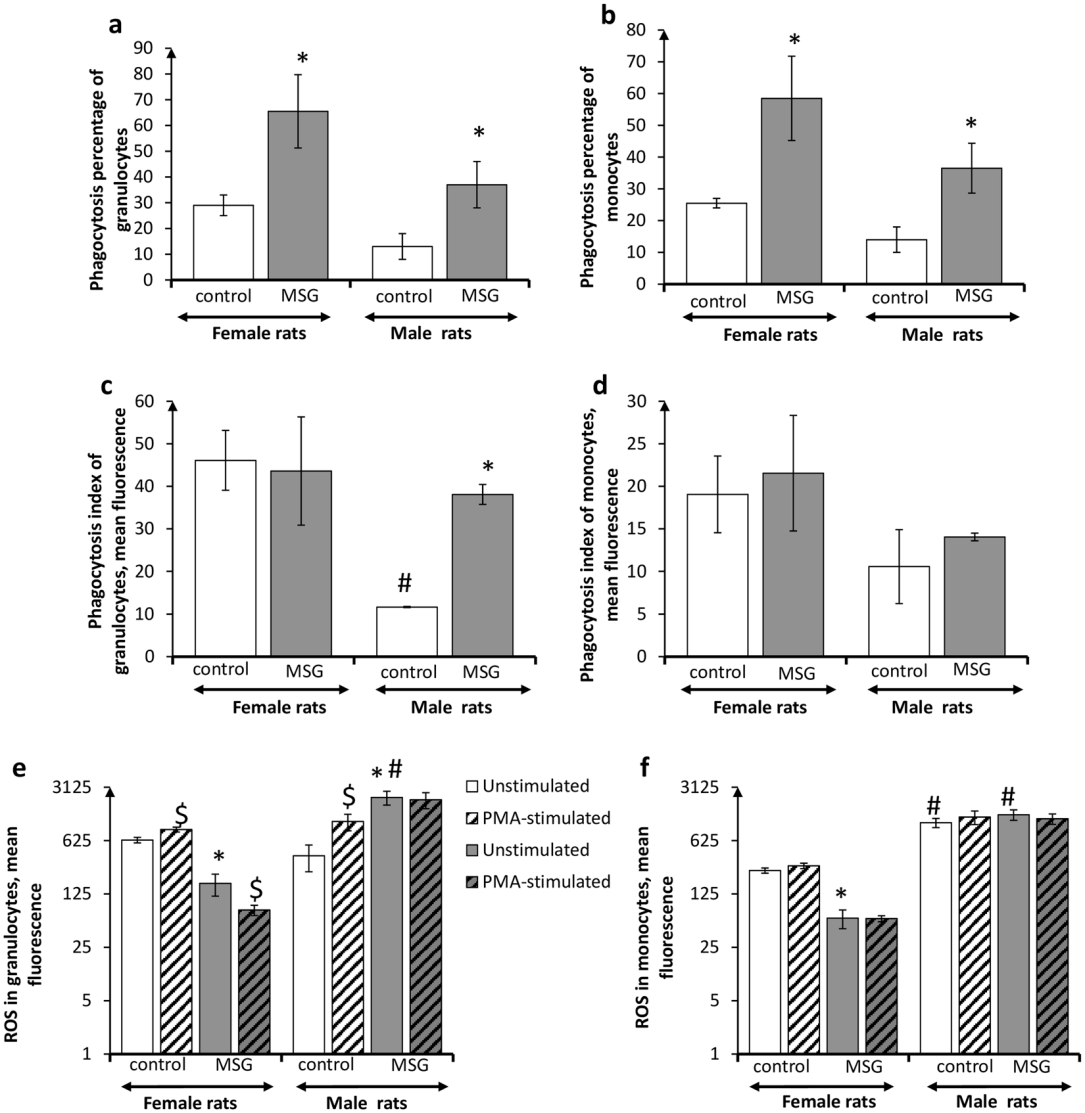
Functional activity of circulating phagocytes in *female* and *male* rats with monosodium glutamate-induced obesity (MSG, n = 8). (**a,b**) Phagocytosis percentage of granulocytes and monocytes, respectively; (**c,d**) phagocytosis index of granulocytes and monocytes, respectively; (**e,f**) intracellular ROS generation by granulocytes and monocytes, respectively. Data are presented as mean ± SD. *P < 0.05 was considered significant compared to the corresponding values of the control animal group. ^$^P < 0.05 was considered significant, compared to the values of the corresponding unstimulated cells. Comparisons between sexes (ANOVA) are shown as follows: ^#^p < 0.05.

Quantitative and functional characteristics of circulating phagocytes in male and female control animals had differences. PhP, PhI and intracellular ROS generation values were significantly higher in females than in males (Fig. 5a–f). Meanwhile, granulocytes from both males and females responded positively to treatment with PMA *in vitro*, whereas monocytes did not respond to this oxidative metabolism stimulator (Fig. 5e,f).

Development of MSG-induced obesity was accompanied by distinct alterations in circulating phagocyte quantitative indices, and metabolic profile in male and female animals. Percentage of circulating phagocytizing monocytes and granulocytes was increased in obese female and male rats, as compared to their non-obese counterparts (Fig. 5a,b). However, phagocytosis intensity in obese females remained unchanged, whereas PhI values of circulating granulocytes in obese male animals were 3.3 times higher than in control animals (Fig. 5c,d). Most profound differences in obese male and female animals were registered in the circulating phagocyte oxidative metabolism. Intracellular ROS-generation in phagocytes from obese females was sharply decreased, as compared to the control animals, whereas in male rats with MSG-induced obesity, circulating granulocytes demonstrated significantly enhanced oxidative metabolism (a 5.8-fold increase) (Fig. 5e,f). The oxidative metabolic reserve of the cells, after stimulation with PMA *in vitro*, was not detected. In the case of male rats, this indicates that oxidative metabolism of circulation phagocytes was activated ad summum.

## Discussion

Despite the numerous approaches to the prevention and treatment of obesity, the prevalence of this pathology, and the various associated disorders keep growing. Obesity related mortality and morbidity differ by sex^4,29^. One of the most important pathogenic factors – in the development of obesity-induced insulin resistance, as well as type-2 diabetes, cardiovascular disease, and other related co-morbidities – is chronic meta-inflammation, which is a constant state of immune system that is characterized by the pro-inflammatory activation of immune cells of different locations^30,31^. Sexual dimorphism is inherent in the functioning of the immune system^9,10^. Despite this fact, the involvement of the sex-based differences in the immune reactivity in the aetiology of obesity, gender inequality remains largely understudied and poorly understood. This concerns, in particular, the neuro-endocrine form of obesity, which is caused by MSG^32^.

We have previously shown that MSG, injected into newborn rats, induces obesity and significant increase of serum level of one of the main pro-inflammatory adipokines – leptin^26,28^. It indicates a systemic pro-inflammatory adipokine profile, which promotes the development of meta-inflammation, and is one of the main reasons for consistent involvement of inflammatory cells, mainly phagocytes, in different locations in this process. This study was aimed at investigating the functional state and metabolic profile of phagocytes from different locations in male and female rats with MSG-induced obesity. Macrophages are well-known resident cells in adipose tissue, and the first to become involved in inflammation associated with obesity. Their number increases in the fat during obesity in humans and animals^33,34^. However, their morphological and functional profile, as well as their potential role in obesity-related diseases, remains controversial. Granulocytes – another population of innate inflammatory cells – are presented in normal adipose tissue by quite low numbers of eosinophils. These cells play an essential role in adipose tissue immunobiology, by promoting alternative polarisation of resident macrophages. The development of obesity shifts the numbers and types of adipose tissue granulocytes. Adiposity is associated with a decline in eosinophile proportion, and with a significant influx of neutrophils^35,36^. Neutrophils in fat tissue maintain inflammation and pro-inflammatory macrophage polarisation. The functional state and metabolic polarization of phagocytes, we characterized by several generally accepted and validated indices, such as the level of CD14 expression, arginine metabolism, oxidative metabolism and phagocytosis activity. A high level of CD14 expression is characteristic for more differentiated phagocytes^37^. The strong association between the obesity-related pathological changes, including inflammation, and CD14 molecules has been demonstrated in rodent models of obesity^38,39^. Moreover, expression of CD14 by pro-inflammatory macrophages is increased, in contrast to the cells with anti-inflammatory phenotype^17^. Arginine metabolisms evaluation is traditionally used to characterize monocyte/macrophage and neutrophil metabolic polarization. Pro-inflammatory (M1, classical) macrophages are characterized by high expression of inducible nitric oxide synthase (iNOS), which utilize L-arginine in reaction of nitric oxide (NO) generation^40–42^. Anti-inflammatory (M2, alternative) phenotype belongs to those cells that are expressing high level of arginase, an enzyme that utilizes L-arginine to generate urea and L-ornithine, which stimulate reparation processes. Previous results from our laboratory, as well as results of other research groups, have shown that ROS generation and phagocytosis activity could also be successfully used as markers of monocyte/macrophage polarization^43–45^. According to these observations, the functional state of anti-inflammatory phagocytes is associated with decreased oxidative metabolism, along with increased endocytosis activity, which is opposite to classically activated cells that demonstrate increased ROS generation, along with inhibited phagocytosis activity. The subsets of neutrophils with different functional activity have been designated as N1 and N2, by analogy with mononuclear phagocytes^46,47^. Similar to alternatively polarized mononuclear phagocytes, N2 neutrophils are characterized by a decreased oxidative metabolism, and promote angiogenesis^48^.

As a result of our experiments, we have shown that the development of MSG-induced obesity in rats was characterized by sex dimorphism. Anthropometric alterations (body weight decrease along with an increase in the Lee index), which accompany the neuro-endocrine form of obesity, were more pronounced in male animals. The accumulation of total fat, as well as distinct divisions of WAT, was also more significant in male rats, than that in female animals. It is necessary to point out, that subcutaneous fat depots were enlarged most dramatically in the male animals. Fat distribution in different adipose tissue areas is one of the major contributing factor in insulin resistance and metabolic disorders in obesity. There is a growing body of evidence, supporting the leading role of subcutaneous adipose tissue in the development of insulin resistance and meta-inflammation in obesity, as this fat depot is a major source of systemic free fatty acid flux^49–51^. Vieira *et al*.^52^ hypothesized that free fatty acids cause a pro-inflammatory skew in the metabolism of resident phagocytes, accompanied by the production of chemokines, cytokines and other systemic inflammatory mediators, which promote systemic propagation of the inflammatory process.

In our experiments, an increased number of CD14+ phagocytes, with low level of this marker expression, was revealed in fat pads of male rats with MSG-induced obesity. We believe that this could be caused by the recruitment of circulating immature cells into inflamed adipose tissue^53,54^. However, the metabolic profile of CD14+ SVF cells, which was evaluated by their phagocytic function, and the level of ROS production, bears evidence in favour of pro-inflammatory activation of these cells in MSG-treated female animals. At the same time, the local inflammatory reaction, in adipose tissue of obese male rats, was rather low-grade.

Phagocytes in the peritoneal cavity closely interact with visceral adipose tissue depots. Their number and functional activity, as well as metabolic polarization, strongly depend on various mediators released by cells of adipose tissue immune cells^14,55,56^. Our data demonstrate sex-associated differences in the functional state of peritoneal phagocytes from rats with MGS-induced obesity. In contrast to the female animals, whose peritoneal phagocytes demonstrated rather a neutral metabolic profile, cells from the male rats exhibited signs of pro-inflammatory metabolic directedness. This indicates the extension of adipose tissue inflammation to the adjacent peritoneal cavity in male rats.

Since obesity is known to induce a state of systemic inflammation^14,33,56^, we also examined the functional state of phagocyte cells in blood samples of rats with MSG-induced obesity. Circulating phagocytes from female obese mice did not exhibit any signs of pro-inflammatory metabolic profile. In contrast, functional indices of circulating phagocytes in male obese rats indicated their pro-inflammatory metabolic profile, especially in granulocytes. The cells were characterized by an increase of both ROS production and phagocytosis activity. Meanwhile, the level of enhancement of oxidative metabolism was considerably higher than that of the phagocytic function in MSG-treated rats. In addition, the cells exhibited no functional reserve after *in vitro* stimulation with PMA. Together, these changes reflect the shift to pro-inflammatory activation of the circulation phagocytes in obese male rats. Thus, the development of glutamate obesity in males is characterized by a generalized inflammatory response of phagocytes of different populations, whereas the inflammatory activation of phagocytes in females is rather localized and limited to resident cells of adipose tissue. Further in-depth research is necessary to elucidate the mechanism and molecular triggers of gender diversification of phagocyte inflammatory activation in MSG-induced obesity.

In conclusion, our study found that the manifestation of inflammation, associated with MSG-induced obesity, is characterized by different patterns of phagocyte metabolic profile, in different locations in male and female rats. The identified phenomenon must be taken into account when monitoring the development of this metabolic disorder. A deep understanding of sex-based differences in immune cell activation in obesity opens the prospect for the development of personalized diagnostic algorithms, and treatment modalities for this pathology and obesity-related diseases.

## Methods

### Wistar rats with MSG-induced obesity

Newborn Wistar female (n = 16) and male (n = 16) rats (bred in the vivarium of the Educational and Scientific Centre “Institute of Biology” of Taras Shevchenko National University of Kyiv, Ukraine) were kept in standard conditions. The male and female rats were divided into two groups of 8 animals each. MSG dissolved in saline was administered to newborn rats of the experimental groups at the dose of 4 mg/g of body weight, with a volume of 8 μl/g subcutaneously on the second, fourth, sixth, eighth and tenth days of life^26^. The newborn rats of the control group were injected with saline at the volume of 8 μl/g subcutaneously at the same time points. The animals were given free access to food and water during 4 months after birth. Body weight and body length (nose-to-anus length) measurements in all groups of rats were made during 4 months from birth. Obesity was determined by the Lee index, which was calculated as the cube root of body weight (g)/nose-to-anus length (cm). Four-month-old animals were sacrificed by decapitation. The rats’ peripheral blood was collected, in tubes containing heparin, for an analysis of circulating phagocytes by flow cytometry. Visceral white adipose tissue (retroperitoneal fat pads, epididymal (perigonadal) fat pads and mesenteric fat pads) and subcutaneous white adipose tissue (inguinal fat pads) were excised and weighed. Total summed fat pad weights and different localization fat pad weights (absolute and relative, % body weight), were used as an index of adiposity^18,26^.

The animal protocol was approved by the Taras Shevchenko National University of Kyiv animal welfare committee, according to the Animal Welfare Act guidelines. The study was conducted in compliance with the standards of the Convention on Bioethics of the Council of Europe’s “European Convention for the Protection of Vertebrate Animals used for experimental and other scientific purposes” (1997), the general ethical principles of animal experiments, approved by the First National Congress on Bioethics Ukraine (September 2001), and national low (LAW OF UKRAINE # 3447-IV) issued by the Cabinet of Ministers of Ukraine (2006). The animals were kept in a vivarium that was accredited, in accordance with the “standard rules on arrangement, equipment and maintenance of experimental biological clinics (vivarium)”. Instruments to be used for research were subject to metrological control.

### Isolation of stromal vascular fraction (SVF) cells from adipose tissue

Total visceral fat pads were used to isolate SVF cells by the standard method^57,58^, with some modifications. Briefly, adipose tissue was weighed, rinsed with PBS, and minced using sterile techniques. Then Hanks buffered saline solution (HBSS) containing 0.1% of collagenase (Sigma-Aldrich) was added to the tissue samples, and the suspensions were incubated for 1 h at 37 °C. After digestion, the suspensions were filtered and centrifuged at 400× g. The pelleted SVF cells were resuspended in PBS, to analyse ROS production and phagocytosis activity by flow cytometry. The total number of cells was counted using trypan blue. Phycoerythrin (PE)- labelled anti-CD14 antibodies (Becton Dickinson, Farmingen, USA) were used to determine the relative amount of CD14+ cells (monocytes and granulocytes), among the SVF cells, and an intensity of CD14 surface expression on the SVF cells (mean fluorescence per cell). The samples were analysed by the FACSCalibur flow cytometer (BD Biosciences, San Jose, CA, USA). The data were analysed using CELLQuest software (BD; Franklin Lakes, NJ, USA).

### ROS release assay

ROS release by the SVF cells and by peritoneal macrophages was assayed by the nitroblue tetrazolium (NBT) reduction method. SVF cells were isolated as described above. Rat peritoneal macrophages (PMs) were isolated by standard method^43^. Cells were centrifuged at 300 g for 5 min at 4 °C, washed twice with HBSS. SVF cells or PMs (2 × 10^5^/well) were incubated in a 5% CO_2_ atmosphere for 1 h at 37 °C in HBSS, containing 1 mg of NBT (Sigma-Aldrich) per ml. The reaction was stopped by the addition of 2 M KOH and 50% dimethyl sulfoxide. The optical density of the formazan was examined in each well at 630 nm, with a plate reader. Each sample was assayed in triplicate, and the results are presented as mean ± SD.

### Nitrite assay

Nitrite level determination was performed, to evaluate NO release into the conditioned media of rat peritoneal macrophages. After 24 h of cultivation, the culture supernatants were collected, and the nitrite concentration in each supernatant was assayed by the Griess reaction^43,59^. Briefly, equal volumes of 2% sulphanilamide, in 10% phosphoric acid and 0.2% naphthylethylene diamine dihydrochloride, were mixed to prepare the Griess reagent. The reagent (100 μL) was added to equal volumes of the supernatant, and the mixture was then incubated for 30 min at room temperature in the dark. The A550 of the formed chromophore was measured with a plate reader. The nitrite content was calculated with sodium nitrite as a standard. Each sample was assayed for nitrite in triplicate. Each value was divided by the number of viable cells, and expressed as nitrite level per 10^6^ cells. The mean value and SD were calculated with normalized values.

### Intracellular ROS assay

ROS levels were measured using 2′7′-dichlorodihydro-fluorescein diacetate (H2DCFDA, Invitrogen), as previously described^43^. Briefly, the SVF cells or PMs were incubated with PBS, containing 10 μM carboxy–H2DCFDA, for 20 min at 37 °C. Heparinized whole blood was incubated with PBS, containing 10 μM carboxy–H2DCFDA, for 30 min at 37 °C to measure ROS production by peripheral blood monocytes and granulocytes. A short recovery time was allowed for the cellular esterases to hydrolyze the acetoxymethyl ester or acetate groups, and render the dye responsive to oxidation. Erythrocytes were lysed with lysis buffer. The cells were then transferred to polystyrene tubes with cell-strainer caps (Falcon, Becton Dickinson, USA) and analysed with flow cytometry (excitation: 488 nm, emission: 525 nm). Only living cells, gated according to scatter parameters, were used for the analysis. Results were presented as mean fluorescence per cell. Phorbol 12-myristate 13-acetate (PMA) (Sigma-Aldrich) was used to evaluate non-specific reserve in phagocytes^44,60^. Reactivity reserve was characterised by the modulation coefficient (MC), which was calculated with the following formula:

$${\rm{MC}}=100-({\rm{S}}\times 100/{\rm{B}}),$$

where S is ROS value in probes stimulated with PMA *in vitro*, B is ROS value in unstimulated probes (basal value).

### Phagocytosis assay

The flow cytometry phagocytosis assay was performed as previously described^43^. Briefly, FITC-labelled heat-inactivated *Staphylococcus aureus* Cowan I bacteria (collection of the Department of Microbiology and General Immunology of Taras Shevchenko National University of Kyiv), at the concentration of 1 × 10^7^ cells/mL in the volume of 5 μL, were added to heparinized whole blood or to the tubes with the SVF cells or PMs. All samples were incubated at 37 °C for 30 min. At the end of the assay, phagocytosis was arrested by the addition of cold stop solution (PBS with 0.02% EDTA and 0.04% paraformaldehyde). Erythrocytes were lysed with lysis buffer. Fluorescence of phagocytes with ingested bacteria was determined by flow cytometry. The results were registered as the percentage of cells emitting fluorescence, after a defined culture period (phagocytosis percentage, PhP), and as phagocytosis index (PhI), that representing the mean fluorescence per one phagocytic cell (ingested bacteria by one cell).

### Statistical analysis

All experimental results are reported as mean ± SD. Two-way analysis of variance (ANOVA) was performed, with factors of sex and obesity. Statistical significance between animal groups was determined by t-test and non-parametric Mann-Whitney U test. χ² test was used for qualitative data. Differences were considered significant at *p* values of 0.05 or less. All data are available from the corresponding author on reasonable request.
